# A High-Sensitivity Flexible Direct X-ray Detector Based on Bi_2_O_3_/PDMS Nanocomposite Thin Film

**DOI:** 10.3390/nano11071832

**Published:** 2021-07-14

**Authors:** Longmei Mao, Yi Li, Hu Chen, Longxin Yu, Jianhua Zhang

**Affiliations:** Key Laboratory of Advanced Display and System Applications of Ministry of Education, Shanghai University, 149 Yanchang Road, Shanghai 200072, China; longmei6996@163.com (L.M.); liyi_shuvip@163.com (Y.L.); chen0305hu@163.com (H.C.); laskyu@shu.edu.cn (L.Y.)

**Keywords:** X-ray detector, flexible, PDMS, Bi_2_O_3_, nanocomposite

## Abstract

The characteristics of mechanical flexibility, low health risk, and simple processing of polymer nanocomposite materials make them potentially applicable as flexible X-ray detectors. In this study, we report on a high sensitivity, environmentally friendly, and flexible direct X-ray detector using polymer nanocomposite material consisting of bismuth oxide (Bi_2_O_3_) nanoparticles and polydimethylsiloxane (PDMS). This detector was realized by printing patterned Ag electrodes on the polymer nanocomposite material. The response of PDMS to X-rays was verified for the first time, and the effect of doping different contents of Bi_2_O_3_ nanoparticles on the performance of the device was tested. The optoelectronic performance of the optimized detector indicated a high sensitivity (203.58 μC Gy_air_^−1^ cm^−2^) to low dose rate (23.90 μGy_air_ s^−1^) at a 150 V bias voltage and the X-ray current density (J_X-ray_) was 10,000-fold higher than the dark current density (J_dark_). The flexible direct X-ray detector could be curled for 10,000 cycles with slight performance degradation. The device exhibited outstanding stability after storage for over one month in air. Finally, this device provides new guidance for the design of high-performance flexible direct X-ray detectors.

## 1. Introduction

X-ray detectors have been widely used in the medical, industrial, and scientific research fields over the past few decades [1,2,3,4,5,6]. From the end of the last century, researchers have gradually turned their attention to direct X-ray detectors as a result of their higher intrinsic X-ray absorption coefficient and spatial resolution than indirect X-ray detectors [7]. Direct X-ray detectors sandwich X-ray sensitive materials between two electrodes and can directly convert X-rays into electrical signals without using scintillator [8]. Amorphous selenium (a-Se) detectors have a relatively wide range of applications in the field of medical imaging [9,10,11]; however, amorphous selenium X-ray detectors still have several major shortcomings, such as high working voltage and low absorption ability under higher energy X-rays [12,13]. In addition, a-Se detectors require a thick film layer to absorb X-rays effectively, which makes them difficult to bend. Therefore, it is necessary to propose a new generation of direct X-ray detector to meet the needs of future development.

High atomic number (Z) materials can attenuate X-rays (40–150 kV) predominantly through the photoelectric effect [14,15]. Generally, organic matters are composed of low-Z materials, which result in low absorption of high-energy photons [16]. In recent years, blends of organic polymer and heavy inorganic nanoparticles, quantum dots or perovskite have been proposed to overcome such issues [14,17,18,19]. Lead [13,20,21,22,23,24] (Pb, Z = 82) is the most widely used X-ray absorbing material commercially, such as a lead chamber or other lead-containing protective clothing. However, long-term exposure to lead or its salts (such as lead oxide (PbO) and lead iodide (PbI_2_)) may cause accumulation of heavy metals in the body, which may lead to serious health problems, such as neuron disease and kidney failure [2]. Therefore, it is necessary to choose a non-toxic and environmentally friendly material. Bismuth [25,26,27,28] (Bi, Z = 83) has also frequently appeared in the research and, recently, for developing X-ray detectors. P. Praveenkumar et al. [29] prepared signal phase Bi_5_O_7_I nanocrystals and studied their performance in X-ray detectors with a sensitivity of 1.924 × 10^−2^ μC Gy_air_^−1^ cm^−^^2^. Imalk Jayawarden et al. [18] dispersed a direct X-ray detector based on Bi_2_O_3_ nanoparticles dispersed in poly(3-hexylthiophene-2,5-diyl) (P3HT) and [6,6]-phenyl C_71_ butyric acid methyl ester (PC_70_BM) with a sensitivity of 3.36 × 10^−2^ μC Gy_air_^−1^ cm^−2^. Here, bismuth oxide (Bi_2_O_3_) was selected as the X-ray sensitive material due to its excellent absorption and attenuation properties for X-rays and its lower health risks [17,30,31].

The range of applications of organic polymers has gradually covered various fields of electronic equipment, such as light-emitting diodes [32], field effect transistors [33], photovoltaic cells [34], and sensors [35], due to their simple manufacturing method and good flexibility. Making X-ray detectors on a flexible substrate produces detectors that have flexible characteristics, which is an important direction for studies on X-ray detectors [36,37]. PDMS is one of the most popular materials in the field of flexible electronics. This material is scalable to large flexible substrates [38] due to its liquid phase, low-temperature, and low-cost deposition techniques. In addition, PDMS is a flexible and environmentally friendly polymer with tunable chemical, physical, and electrical properties and it is usually used in biomedical and in vivo applications [39,40]. There are also active layers of some electronic devices that use a combination of nanomaterials and PDMS. Massaro et al. presented a millimeter pillar-type sensor made of gold micro/nanoparticles in PDMS material [41]. Wang et al. realized a flexible nanogenerator based on P(VDF-TrFE) nanofibers and PDMS/carbon nanotubes thin composite membrane, which worked under triboelectric and piezoelectric hybrid mechanisms [42]. Sriphan et al. presented a high-performance hybridized nanogenerator that operated from the composite film of Ti_0.8_O_2_ nanosheets/silver nanoparticles co-doped BaTiO_3_ nanopowders inside the PDMS host [43]. However, there are few related studies on the X-ray response of semiconductor nanomaterials and PDMS composites.

In this study, we dispersed Bi_2_O_3_ nanoparticles in PDMS uniformly and printed patterned silver electrodes on the surface of the film to fabricate a flexible polymer nanocomposite membrane direct X-ray detector. To optimize the sensitivity of the X-ray detector, we considered flexible thin-film X-ray detectors with different weight percentages of Bi_2_O_3_ nanoparticles in PDMS and different thicknesses. The 70 weight ratio %, among the Bi_2_O_3_ to PDMS weight ratios, exhibited the optimum harvesting performance among all the compositions, with the highest sensitivity of 203.58 μC Gy_air_^−1^ cm^−2^ at a bias voltage of 150 V under a dose rate of 23.90 μGy_air_ s^−1^. In addition, the device exhibited excellent flexibility with almost the same photocurrent after 10,000 times crimp tests and a stable response after 30 days in an atmospheric environment.

## 2. Materials and Methods

### 2.1. Materials

All chemicals and solvents were purchased from commercial suppliers and used as received. Sylgard 184 polydimethylsiloxane elastomer base and curing agents were obtained from Dow Corning Corporation (Midland, MI, USA) and mixed at a recommended ratio of 10:1 before using. Bi_2_O_3_ nanoparticles were purchased from Alfa Aesar (Ward Hill, MA, USA). Ag ink was purchased from Beijing Dahua Boke Intelligent Technology Co., Ltd. (Beijing, China). Polyimide (PI) was made in the laboratory.

### 2.2. Device Fabrication

**Figure 1:**
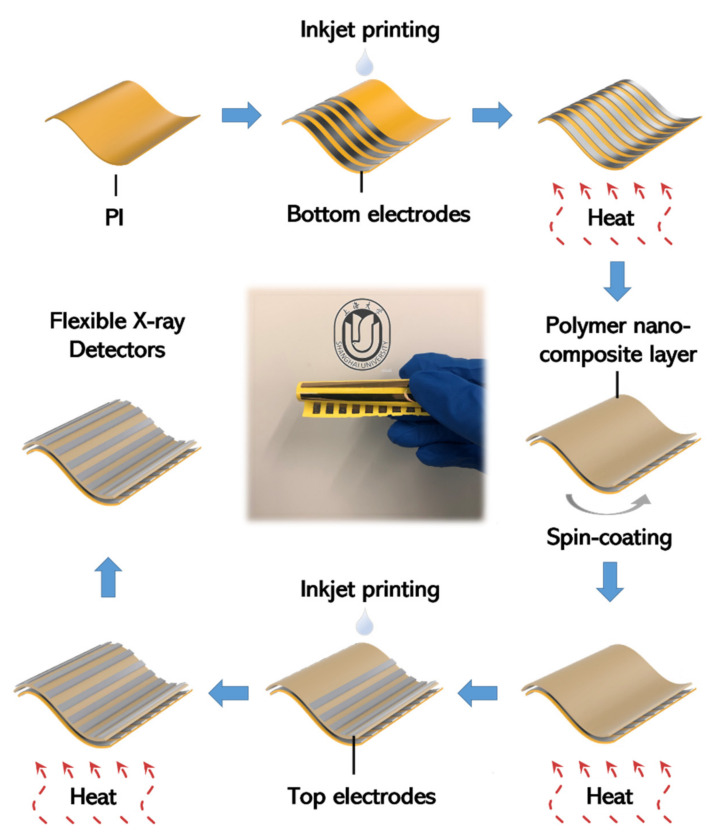
The schematic diagram of the device fabrication.

The schematic diagram of the device fabrication is shown in Figure 1, and the detail process is described as follows:

**Bottom electrodes** The PI was cleaned with deionized water for 10 min and dried with compressed air before using. The bottom electrodes were printed on the PI substrate. In the printing process, the substrate temperature was set at room temperature, and the Ag ink was jetted with a 30 μm dot pitch and only one nozzle was used during the printing process. Two layers were printed consecutively to ensure good continuity and conductivity of the printing electrodes. Then, the substrate was baked at 150 °C for 30 min. The square resistance of the bottom electrode was about 450 mΩ/cm^2^.

**Polymer nanocomposite layer** Different masses of Bi_2_O_3_ nanoparticles were added into the 2 g PDMS samples, respectively, and stirred for 5 min to obtain polymer nanocomposite slurries with different weight ratios (weight ratio %) of 10 (β-10%), 30 (β-30%), 50 (β-50%), 70 (β-70%), 90 (β-90%), and 110 (β-110%), respectively. The concentrations of Bi_2_O_3_ are listed in Table 1**.** The weight ratio used in this work is defined as:

$$wt\%\;of\;Bi_{2}O_{3}=\frac{Weight\;of\;Bi_{2}O_{3}\;}{Weight\;of\;PDMS}\times 100\%$$

Then, the polymer nanocomposite slurries were spin coated on the bottom electrodes and the samples were cured at 80 °C for 2 h.

**Top electrodes** The surface of the polymer nanocomposite layer was hydrophobic and could not provide suitable wettability for the Ag ink. In this study, we used an oxygen plasma treatment (250 W for 20 s) to modify the surface of the polymer nanocomposite layer to be hydrophilic. Then, the top electrodes were printed on the polymer nanocomposite layer with a 50 μm dot pitch and only one nozzle was used during the printing process. Two layers were printed consecutively to ensure good continuity and conductivity. Finally, the samples were baked at 150 °C for 30 min. The square resistance of the top electrode was about 750 mΩ/cm^2^.

### 2.3. Characterization

All the measurements were carried out in air at room temperature using an active device area of 0.09 cm^2^. A semiconductor Parameter Analyzer (PDA, FS-380, PSAIC, Beijing, China) was used to measure the current-voltage characteristics, with a tube X-ray beam (Moxtek MADPRO, MOXTEK, Orem, UT, USA) with accelerating voltage of 60 kV under a dose-rate range of 0.023–11.97 mGy_air_ s^−1^. Dose calibration was completed using a radiation dosimeter (IBA, Magic MaX Universal, Wuhan, China) in a lead chamber. Inkjet printing was performed with a Fujifilm Dimatix DMP-2831 (FUJIFILM Dimatix Inc., Santa Clara, LA, USA), assembled with a 10 pL rated droplet volume ink cartridge with a 16-nozzle piezoelectric printhead. All printing processes were performed at a jetting frequency of 5 kHz. A planetary mixer (ZYMC-580, Shenzhen, China) was used to mix the Bi_2_O_3_ nanoparticles and PDMS.

The X-ray diffraction (XRD) was carried out on a Rigaku MinFlex 6G system (Tokyo, Japan). The scanning electron microscope (SEM) experiments were obtained using a ZEISS Sigma 300 (Jena, Germany). The transmission electron microscopy (TEM) images were obtained using a Tecnai G2 F20 (FEI, Hillsboro, OR, USA). The electronic differential system (EDS) analysis was carried out using an X-MaxN Oxford Instrument system (Oxford, UK). The thermogravimetric analysis (TGA) was carried out using a Mettler Toledo LF system (Zurich, Switzerland), in a temperature range of 25–650 °C under nitrogen atmosphere, at a heating rate of 10 °C/min.

## 3. Results and Discussion

**Figure 2:**
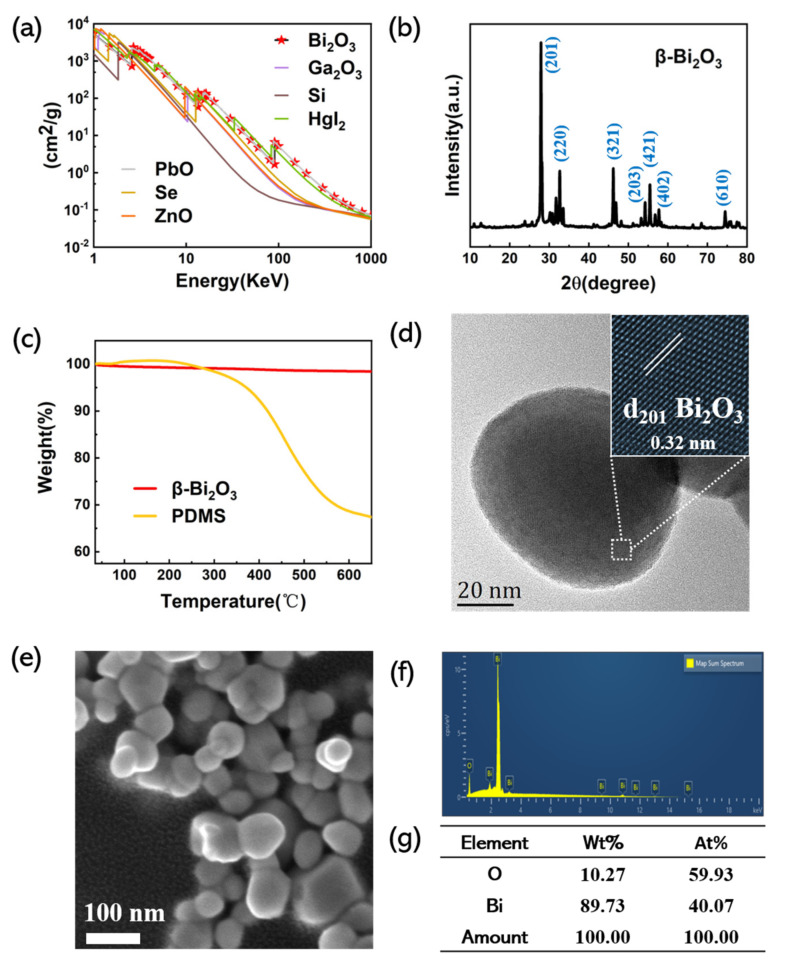
(**a**) Absorption coefficients values of Bi_2_O_3_, Ga_2_O_3_, Si, HgI_2_, PbO, Se, and ZnO; (**b**) the XRD pattern; (**c**) the TGA of Bi_2_O_3_ nanoparticles and PDMS; (**d**) a TEM image of Bi_2_O_3_ nanoparticles, inset is high-resolution TEM image and shows the d-spacing of 0.32 nm correlating to the (201) plane; (**e**) a SEM image of Bi_2_O_3_ nanoparticles; (**f**,**g**) the EDS of Bi_2_O_3_ nanoparticles.

The linear attenuation coefficients of Bi_2_O_3_ and several typical semiconductors in the energy range of 1−1000 keV were calculated based on the data from the National Institute of Standards and Technology, as shown in Figure 2a. A typical XRD pattern of the sample is shown in Figure 2b and exhibits the characteristic diffraction peaks indexed to (201), (220), (321), (203), (421), (402), and (610). All observed XRD patterns match with the standard (JCPDS 27–0050) of pure β-Bi_2_O_3_ nanoparticles. Figure 2c shows the TGA of Bi_2_O_3_ nanoparticles and PDMS. The weight loss curve of PDMS shows a single step weight reduction of 38.2% between 341 and 566 °C. This was due to the loss of methyl groups on the Si-O backbone.The β-Bi_2_O_3_ nanoparticles have excellent thermal stability with almost no loss during the test. Figure 2d shows the TEM image of highly crystalline β-Bi_2_O_3_ nanoparticles. The high-resolution TEM reveals lattice fringes corresponding to the (201) plane of β-Bi_2_O_3_ (Figure 2d inset), in agreement with the XRD spectra. In Figure 2e, the SEM image provides an overall view of the β-Bi_2_O_3_ nanoparticles. An average size of ~40 nm can be observed for β-Bi_2_O_3_. Figure 2f,g is the EDS of β-Bi_2_O_3_ nanoparticles and indicates the element stoichiometric ratio of Bi and O is 2:3.

The X-ray detector developed in this work was based on PDMS and β-Bi_2_O_3_ nanoparticles. In order to optimize the detector’s performance, the concentration of β-Bi_2_O_3_ nanoparticles in the polymer nanocomposite layer was increased for increased X-ray attenuation (10, 30, 50, 70, 90, and 110 weight ratios %; X weight ratio % is noted as β-X%).

**Figure 3:**
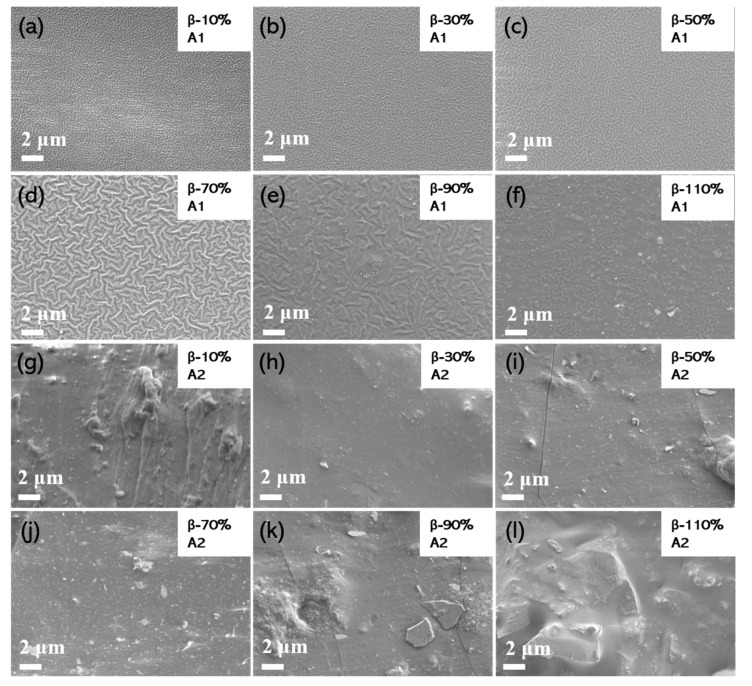
SEM images of polymer nanocomposite layers with different weight percentages. (**a**–**f**) The surface of polymer nanocomposite layers with different weight percentages. (**g**–**l**) The cross-section of polymer nanocomposite layers with different weight percentages. A1 and A2 mean the surface and cross-section of the layers, respectively.

It is important to analyze the surface morphology of organic materials. From the micrographs, the nature of the surface, particle distribution, and surface failure can be observed. The surface microstructure and roughness can be easily studied by SEM, and the wettability of the surface is usually affected by the microstructure and roughness on the surface [44]. In Figure 3a–f, the SEM images demonstrate the surface morphology of the polymer nanocomposite layers as the weight percentage increases. It can clearly be seen that the surfaces of polymer nanocomposite layers changed from pleated to flat as the weight percentage increases. In Figure 3a, there are lots of wrinkles all over the surface. When the weight percentage increases to 110%, the surface morphology becomes flatter than the others. The difference in surface morphology may be attributed to the influence of the different doping contents of nanoparticles on the structure of PDMS [45]. In Figure 3g–l, the SEM images show the cross-section of the polymer nanocomposite films with different weight rates. As shown in Figure 3g–j, the β-Bi_2_O_3_ nanoparticles were evenly dispersed in the PDMS. However, the β-Bi_2_O_3_ nanoparticles in β-90% and β-110% were agglomerate (Figure 3k–l). This uneven material distribution may have a negative impact on the transfer of charges.

**Figure 4:**
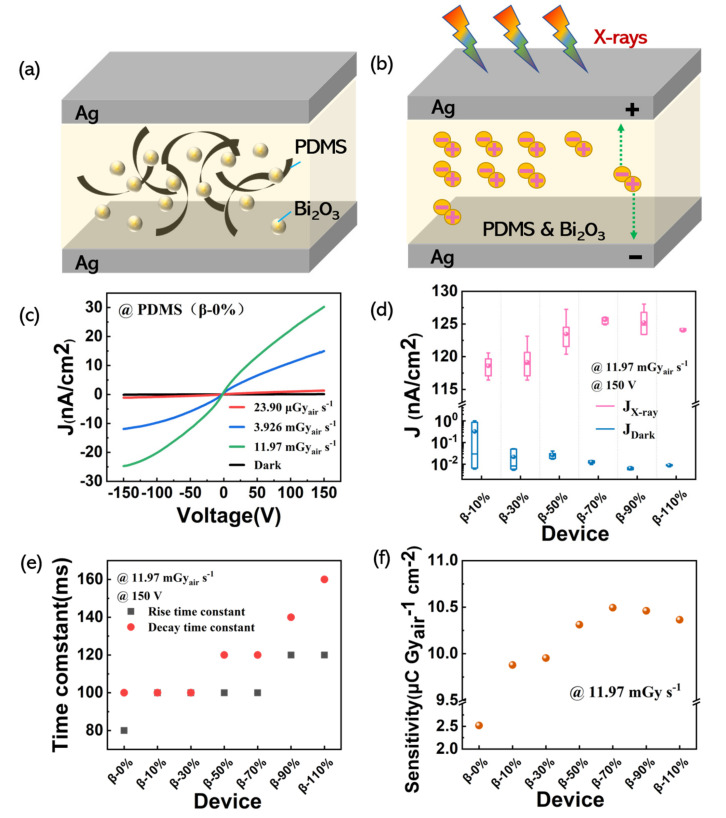
(**a**) The schematic of the device structure; (**b**) a diagram of the process of the conductivity induced by X-ray exposure of the detector; (**c**) the I-V curve of PDMS under different dose rates; (**d**) the X-ray and dark current density box plot of different devices and the error bars represent the range of the current density values; (**e**) rise and decay time constants for detectors with increasing β-Bi_2_O_3_ nanoparticle loadings; (**f**) the sensitivity values for seven devices at 150 V bias voltage.

The schematic of the device structure is shown in Figure 4a. The polymer nanocomposite layer contains β-Bi_2_O_3_ nanoparticles and PDMS is sandwiched between two Ag electrodes. When the X-rays are irradiated, electrons and holes are generated in the polymer nanocomposite layer, and they move to the positive and negative electrodes, respectively, under the influence of the electric field to form a current (Figure 4b). To evaluate the performances of the devices, we expose detectors under an X-ray source to measure the electrical signal including the dark current density, X-ray current density and rise, and decay time constants in Figure 4c–f.

First, we explored the X-ray detection performance of pure PDMS. In Figure 4c, the X-ray current density of PDMS is exhibited under different dose rates of X-rays (from −150 to 150 V). In order to study the electrical properties of the detector under different weight ratios of β-Bi_2_O_3_ nanoparticles, we measured the photocurrent density and dark current density of the detector. Figure 4d shows the photocurrent density and dark current density of different devices. The box plot clearly shows that the photocurrent density begins to decrease after the β-70% device reaches the highest value as the content of nanoparticles increases. The dark current density starts to increase after the β-90% device reaches its minimum value. Figure 4e shows the rise time (to 90% of the maximum signal) and the decay time (to 10% of the maximum) as a function of the devices with different weight percentages. The slow response (>100 ms) is due to the trap states generally present in metal oxide surfaces which impede charge transfer [46].

The sensitivity (S) of a material is a key parameter for X-ray detectors because it can allow the material to easily detect a small X-ray dose or, in other words, it reduces ionizing radiation risks [17]. The definition of sensitivity is the linear dependence of the X-ray photocurrent signal $∆I=I_{X-ray}-I_{dark}$ as a function of impinging dose rate:

$$S=\frac{I_{X-ray}-I_{dark}}{A\times D}$$

where $I_{X-ray}$ and $I_{dark}$ are the current under X-ray irradiation and in the dark, respectively, *D* is the dose rate, and *V* is the active detecting area.

The sensitivity of different devices under 11.97 mGy_air_ s^−1^ at 150 V bias voltage are shown in Figure 4f. The sensitivity of pure PDMS is about 2.51 μC Gy_air_^−1^ cm^−2^. The highest sensitivity of the detectors with different β-Bi_2_O_3_ weight ratio is 10.49 μC Gy_air_^−1^ cm^−2^ (device β-70%) under 11.97 mGy_air_ s^−1^ at 150 V bias voltage.

**Figure 5:**
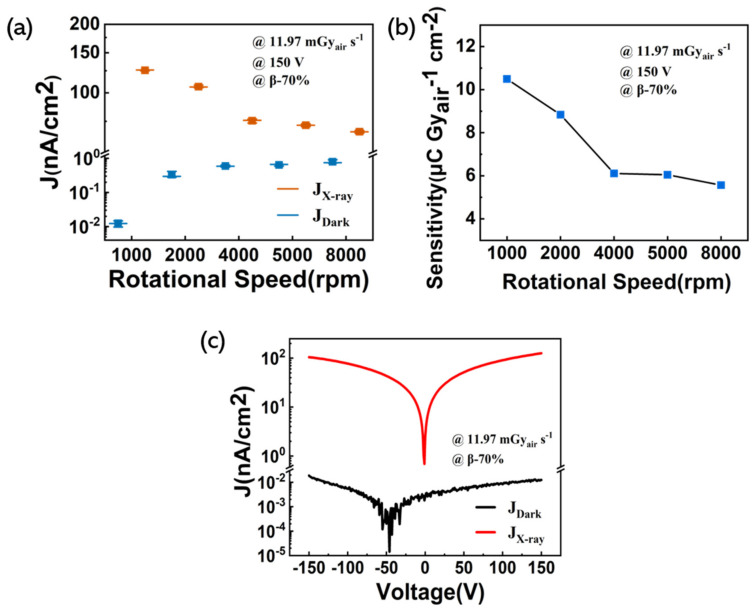
(**a**) The X-ray and dark current density of device β-70% with different thicknesses; (**b**) the sensitivity of device β-70% with different thicknesses; (**c**) I−V characteristics of the β-70% detector (1000 rpm) in the dark and under X-ray irradiation on a logarithmic scale.

After the previous discussion, we found that the β-70% detector had the highest sensitivity among the devices with different nanoparticle contents. In order to further optimize the performance of the device, the thickness of the β-70% detector was varied by changing the spin coating rotational speed. Table 2 lists the thicknesses of the polymer nanocomposite layer at different speeds. Figure 5a shows the X-ray current density and dark current density of different thickness devices. The dark current density behavior is speculated based on the decreased resistance between the top and bottom electrodes as the rotational speed increases. The X-ray current density decreased as the thickness decreased. This may be due to a decrease in the number of charges generated by the polymer nanocomposite film under the same dose of X-ray radiation as the film thickness decreases.

Figure 5b shows the sensitivity as a function of the different thicknesses of devices. When a 290 µm thick (1000 rpm) polymer nanocomposite device was measured under 60 keV X-rays with a bias voltage of 150 V, the sensitivity was up to 10.49 µC Gy_air_^−1^ cm^−2^. Figure 5c shows the I−V characteristics of the β-70% detector (1000 rpm) under X-ray irradiation with a dose rate of 11.97 mGy_air_ s^−1^. The J_X-ray_ is 10,000-fold higher than the J_dark_ at 150 V bias voltage, indicating a high photo-to-dark current ratio of the detector.

**Figure 6:**
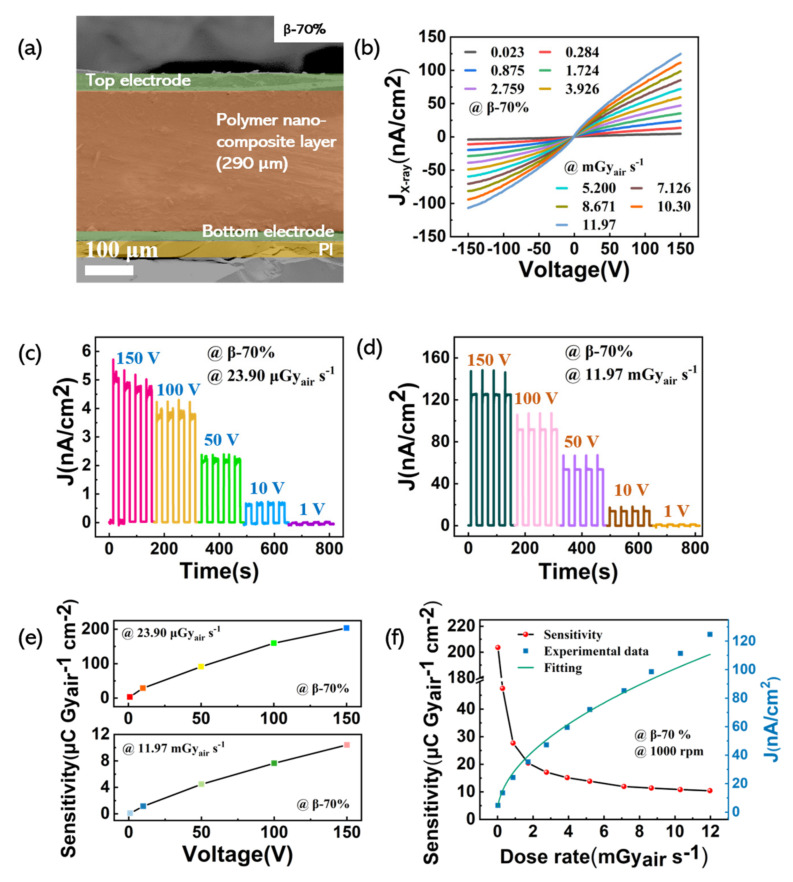
The performance of the β-70% detector (1000 rpm): (**a**) The cross-section SEM image; (**b**) J−V curves of the detector under X-ray irradiation with different dose rates; (**c**,**d**) transient response of the detectors under X-ray irradiation with different dose rates; (**e**) the sensitivity of the detector at different bias voltages; (**f**) the sensitivity and X-ray current density of the detector at different dose rates with 150 V bias voltage.

In Figure 6a, cross-sectional analysis by SEM shows the different layers of the detector. The β-70% polymer nanocomposite layer (1000 rpm) was evaluated in a device with a structure composed of PI (35 μm)/bottom electrode (20 μm)/polymer nanocomposite layer (290 μm)/top electrode (35 μm). Figure 6b shows the J_X-ray_ of the detector under X-ray irradiation with the dose rate in the range of 0.023–11.97 mGy_air_ s^−1^. It is obvious that all the J_X-ray_ increase with the increasing X-ray dose rate. In Figure 6c,d, the transient response of the detector was measured as the X-ray tube was switched between on and off states with a dose rate of 11.97 μGy_air_ s^−1^ and 23.90 mGy_air_ s^−1^ at different bias voltages. No matter under different bias voltages or different dose rates, the detector exhibited a fast response and excellent repeatability. Figure 6e shows that the sensitivity increased monotonically with the increasing bias voltage. The X-ray generated charges can rapidly transport to the electrodes with a low recombination possibility at a high bias voltage, leading to enhanced carrier collection efficiency [47]. The sensitivity of the β-70% detector (1000 rpm) was increased to 203.58 μC Gy_air_^−1^ cm^−2^ under a dose rate of 23.90 μGy_air_ s^−1^ at a 150 V bias voltage.

Figure 6f shows the dose rates as a function of the J_X-ray_ and the sensitivity. Laura Basirico et al. proposed that the nonlinearity between the J_X-ray_ and the dose rates may come from the photoconductive gain [48]. The radiation time determines the sensitivity of the organic X-ray detector, due to the existence of the photoconductive gain, i.e., the longer the exposure time, the sensitivity shows the maximum at low dose rates, and shows a nonlinear relationship with the increasing dose rate. During our test, we exposed our device to X-rays for more than 100 s to obtain the average X-ray current density. Thus, a sublinear relationship between the J_X-ray_ and the increasing dose rate could clearly be observed. To evaluate the sensitivity of the β-70% detector (1000 rpm), the slope of the device output J_X-ray_ versus X-ray dose rate was calculated. The highest experimental sensitivity was 203.58 μC Gy_air_^−1^ cm^−2^ and was obtained at a low dose rate of 23.90 μGy_air_ s^−1^ and 150 V bias voltage. If we take the layer thickness into account, such as done in [17,49], the sensitivity value can be 702 μC Gy_air_^−1^ cm^−3^. We further compared the sensitivity value of our device with the detectors provided in the literature that had the same structure type as our device, as shown in Table 3. Our detector shows better sensitivity than similar types of devices in the literature, and it also provides a potential possibility for further exploration of flexible, non-toxic, and environmentally friendly X-ray detectors in the future.

**Figure 7:**
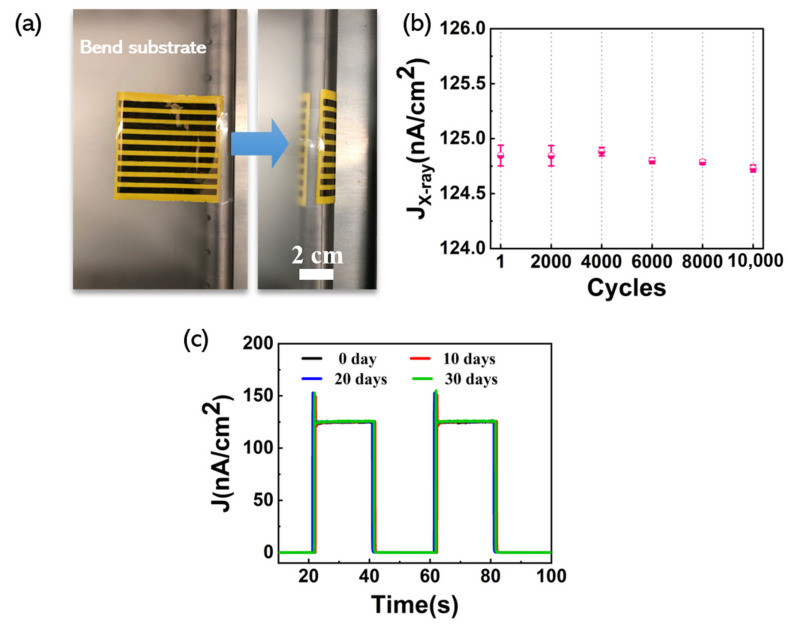
(**a**) Experimental set-up for testing the flexibility of the devices (bending radius is 1 cm); (**b**) J_X-ray_ of the devices measured after 1, 2000, 4000, 6000, 8000, and 10,000 curling cycles; (**c**) time-dependent J of the detector measured every 10 days. The data displayed in Figure 7 was measured under a dose rate of 11.97 mGy_air_ s^−1^ at 150 V bias voltage and in flat-substrate condition.

To evaluate the mechanical flexibility and stability of the β-70% detector (1000 rpm), we characterized the detectors in a curly configuration, and we also re-tested the time-dependent response performance every 10 days. Figure 7a shows the experimental set-up for testing the flexibility of the devices, and the bending radius is set to 1 cm. The detector array was pasted on the bending substrate and curled onto the surface of the metal rod by mechanical movement. In Figure 7b, it shows the J_X-ray_ measured under a dose rate of 11.97 mGy_air_ s^−1^ at 150 V bias voltage after curling and as a function of the number of curling cycles applied to the devices. The error bars refer to the fluctuation of the signal amplitude of the devices. It can be clearly seen that the J_X-ray_ decreases slightly with an increase in the number of curling cycles. Figure 7c shows the stability of the detector stored in air. We repeatedly measured the time-dependent response performance of the device four times in one month. Three on/off switching cycles (20 s for each state) of the X-ray in the same condition were tested, and we extracted the last two cycles to assess the stability. In these numerous tests, there was almost no change found in the J.

## 4. Conclusions

In conclusion, a flexible direct X-ray detector based on Bi_2_O_3_ nanoparticles in PDMS with high sensitivity was successfully investigated. We discussed the performance of polymer nanocomposite layers with different contents of Bi_2_O_3_ NPs and optimized the thickness to obtain the optimal X-ray detector (β-70% detector, 1000 rpm). The device has a consistent response at different dose rates, and the J_X-ray_ is 10,000-fold higher than the J_dark_ at a 150 V bias voltage, which proves that the device has excellent detection performance. The highest sensitivity (203.58 μC Gy_air_^−1^ cm^−2^ in β-70% detector) was achieved under a dose rate of 23.90 μGy_air_ s^−1^ at 150 V bias voltage. Additionally, no significant degradation was observed during the bending and stability test. These results provided guidance for the next-generation high-performance flexible direct X-ray detectors.

## Figures and Tables

**Table 1:** Concentrations of Bi_2_O_3_.

Sample Name	Bi_2_O_3_ in the Dry Film	
	Weight Ratio %	Weight Percent %
β-10%	10	9.1
β-30%	30	23.1
β-50%	50	33.3
β-70%	70	41.2
β-90%	90	47.4
β-110%	110	52.4

**Table 2:** Correspondence between spin coating speed and thickness.

Rotational Speed (rpm)	1000	2000	4000	5000	8000
Thickness (μm)	290	100	52	40	16

**Table 3:** The performances of the X-ray detector in this study as compared with similar detectors reported in the literature.

Materials	Sensitivity (μC Gy_air_^−1^ cm^−2^)	Voltage (V)	Thickness (μm)	Dose Rate ^1^ (μGy_air_ s^−1^)	Reference
CsPbBr	17.7	0.1	1000	17.2	[50]
a-Se	20	2000	200	-	[9]
β-Ga_2_O_3_	66	−15	400	695	[51]
PTAA:Bi_2_O_3_ NPs	0.4 ^2^	−200	20	13,000	[14]
TIPS-pentacence	4.8	−3	0.1	6500	[52]
PFO:Bi_2_O_3_ NPs	24	−80	1	20,000	[19]
PDMS:Bi_2_O_3_ NPs	203.58	150	290	23.90	this work

^1^ The minimum dose rate used when testing the device in the article. ^2^ Not mentioned in the article, estimated based on existing data.

## Data Availability

The data presented in this study are available on request from the corresponding author.
